# Automated classification of breast cancer morphology in histopathological images

**DOI:** 10.1186/1746-1596-8-S1-S29

**Published:** 2013-09-30

**Authors:** Ville Ojansivu, Nina Linder, Esa Rahtu, Matti Pietikäinen, Mikael Lundin, Heikki Joensuu, Johan Lundin

**Affiliations:** 1 Institute for Molecular Medicine Finland-FIMM, Helsinki, Finland; 2 University of Oulu, Center for Machine Vision Research, Oulu, Finland; 3 Department of Oncology, Helsinki University Central Hospital, Helsinki, Finland

## Background

The morphology of a breast cancer tumour, as examined through an optical microscope, is currently assessed visually by the pathologist in parallel with making the cancer diagnosis. The grade of differentiation, which describes how closely the morphology of the tumour resembles the corresponding healthy tissue of an organ, is undisputedly related to the outcome of breast cancer [1]. However, tumour grade is largely regarded as an unreliable prognostic factor due to its poor reproducibility [2]. The visually determined morphology is afflicted with a poor inter- and intra observer agreement, which prevents grade from being fully utilized as an important outcome predictor. The same pathologist may assign different grade to the same tumour when assessment is repeated, and different pathologists disagree to a substantial level when assessing the same tumour [3].

Computational diagnostic tools for estimating the morphological properties of cancer tissue would enable objective and reproducible alternative for diagnosis. This could be achieved by fully utilizing the recent advances in digital microscopy and computer vision [4,5]. Some attempts have already been made for automated grading of histopathological breast cancer images, but these studies have covered only limited amount of data or produce just a partial grading [6,7]. We propose a texture based algorithm for automated classification of breast cancer morphology. The method uses the recently introduced LPQ [8] as well as LBP [9] descriptors and an SVM classifier. The LPQ and LBP descriptors each form a histogram representing the statistical texture properties and have been used earlier in many texture analysis applications which include surface inspection [9], tissue analysis [5], and face recognition [8], whereas SVM represents the state of the art among supervised learning based classification algorithms.

## Material and methods

**Figure 1:**
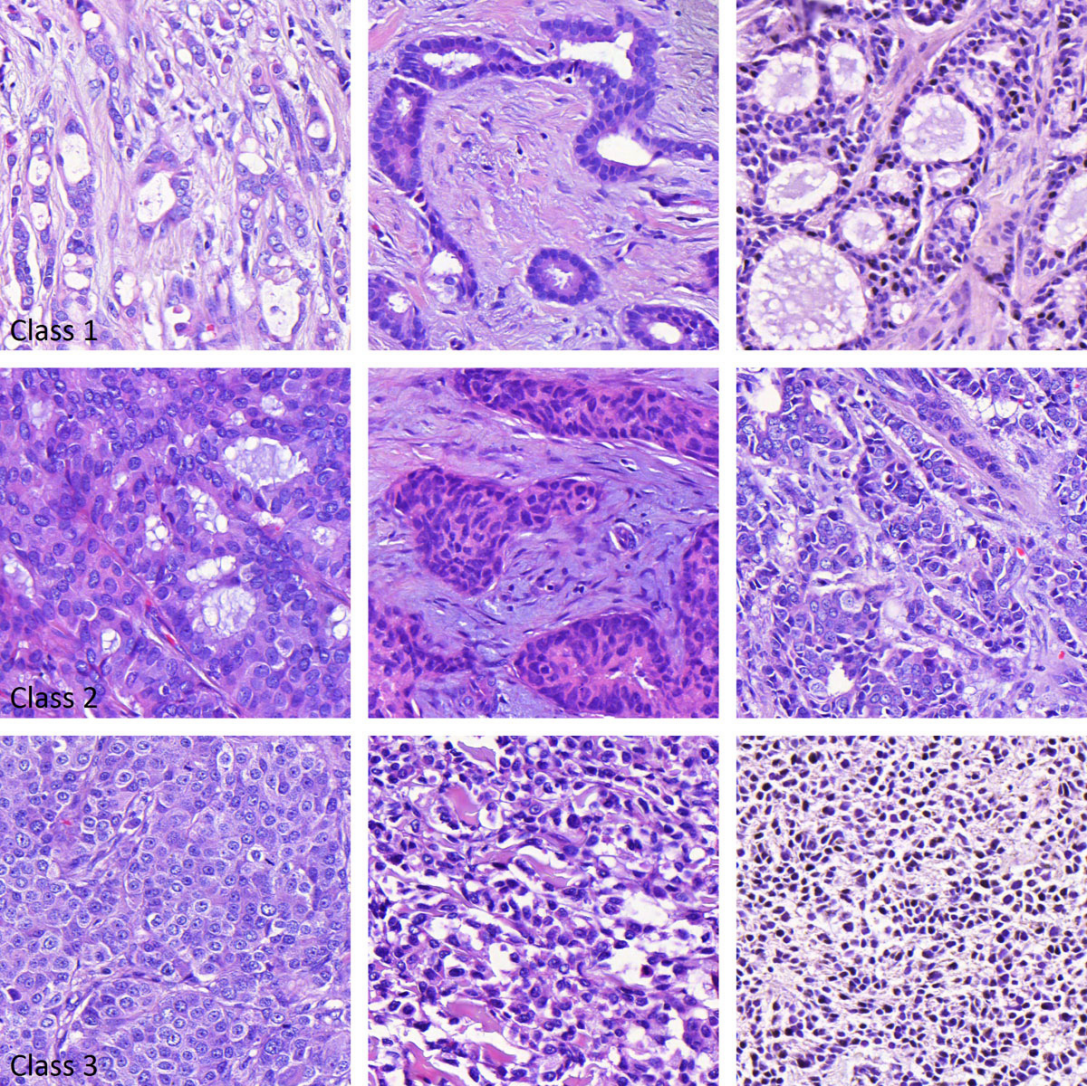
**Examples of tissue images from three morphological classes** Class 1 (top row): morphology resembling normal breast epithelium, extensive tubular formation, n=182; Class 2 (center row): intermediate tubular formation, n=494; and Class 3 (bottom row): morphology least resembling normal breast epithelium, no tubular formation, n=416. Images are classified into the three classes by a human observer.

The image data set (n=1092) was extracted from a series of digitized, whole-slide tissue microarray (TMA) samples from a nationwide cohort of breast cancer patients, FinProg [10]. A single continuous area that contains only tumor tissue was defined in each representative tissue spot in the hematoxylin-eosin (HE) stained TMA samples. The original tissue spots fit into an approximately 1600 x 1600 pixel image while the size of the defined square areas was varying with dimensions in the range 400–1400 pixels. The images were scored by a human observer into three classes according to morphology: 1 (morphology resembling normal breast epithelium, extensive tubular formation, n=182), 2 (intermediate tubular formation, n=494), and 3 (morphology least resembling normal breast epithelium, no tubular formation; n=416). Examples of the three classes are illustrated in Figure 1.

The images were transformed to gray scale and represented by LBP [7] and LPQ [8] texture descriptors. The classification of the images into the three classes was done using three one-versus-rest SVM classifiers with a radial basis function kernel (RBF) combined with chi-square distance metric. The final class was chosen by selecting the largest of the scores produced by the individual SVM classifiers. Given the training samples and their classes, an SVM classifier learns a model for the data which aims to separate the classes in space with a margin. In testing phase, the SVM classifier assigns new data samples into the classes based on the learned model. In our experiments, the data was split into two halves for training and testing of the SVM classifiers. We did additional experiments with only the extreme class 1 and 3 samples. In this case, we used the same descriptors and a binary SVM classifier with an RBF kernel.

## Results and discussion

**Figure 2:**
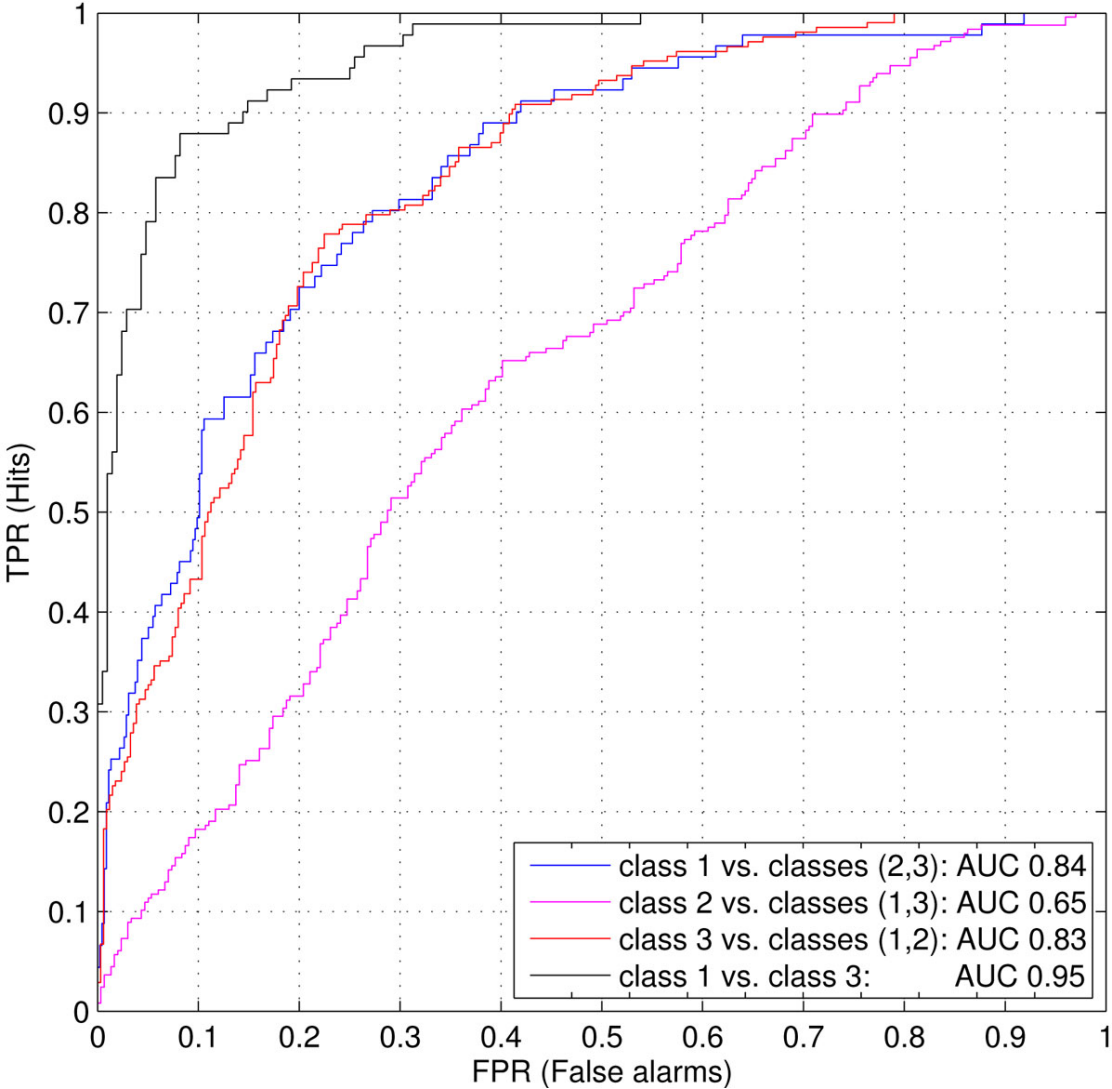
**ROC curves showing the classification performance** Receiver operating characteristic (ROC) curves for each of the three one-vs-rest SVM classifiers. Fourth ROC curve is for an SVM classifier separating classes 1 and 3. Also the relative areas under the ROC curves (AUC) are denoted which express the fidelity of the classification result.

The experiments were performed using different combinations of LBP and LPQ descriptor variants as well as by various scales of the images. The best classification results were achieved by combining the basic versions of LPQ and LBP descriptors with radius r=1 and number of samples p=8 into a 512-dimensional feature vector and using the original image scale 1:1. The receiver operating characteristic (ROC) curves illustrated in Figure 2, show the ratio of the “true positive” and “false positive” samples in classification when the threshold for each binary one-vs-rest SVM-classifier score is changed. The area under the ROC curve (AUC) is related to the fidelity of the classification result. The AUCs for the ROC curves were: class 1 (extensive tubule formation) vs. classes {2, 3}, 0.84; class 2 (moderate tubule formation) vs. classes {1, 3}, 0.65; and for class 3 (no tubule formation) vs. classes {1, 2}, 0.83. If each image is classified into the class with the highest SVM score, the total classification accuracy is 62.0 %. The total classification accuracy was improved by 2 % by using the LPQ descriptor in addition to the traditional LBP descriptor. It seems that the separation of intermediate class 2 from the classes 1 and 3 is the most challenging task. This is understandable since image content in class 2 samples is a mixture of the two neighbouring classes 1 and 3. If it would be enough to separate only the extreme morphological classes 1 and 3 neglecting the class 2, a single binary SVM classifier could be used. For this class 1 vs. class 3 classifier AUC is 0.95 which is remarkably better than the results for the one-vs-rest classifiers. The accuracy of class 1 vs. class 3 classifier is 90 % (when threshold=0 for SVM score is used). One option for better separation of class 2 could be to do the analysis for smaller image areas which would be classified as class 1 or 3. Then class 2 could be found as an appropriately selected mixture of these areas.

## Conclusions

Histological grade of breast cancer is regarded as an important prognostic factor, but not included in staging guidelines due to the subjective nature of the assessment process. In the current study, we propose a computer vision method based on texture features and a classifier utilizing supervised machine learning to discriminate between cancer morphology as determined by a human observer. The results obtained show that automated grading is feasible and that discrimination between different levels of tubule formation can be performed with moderate to high accuracy. By combining LBP and LPQ features it is possible to improve the discrimination accuracy compared to using only LBP alone. While the extreme morphological structures according to tubule formation in the breast cancer tissue are discriminated with high accuracy, the recognition of the intermediate class should still be improved.

## List of abbreviations used

AUC: Area under the ROC curve; HE: Hematoxylin-eosin; LBP: Local binary pattern; LPQ: Local phase quantization; RBF: Radial basis function; ROC: Receiver operating characteristic; SVM: Support vector machine; TMA: Tissue microarray

## Competing interests

The authors declare that they have no competing interests.

## Authors' contributions

VO designed, implemented, and tested the classification system and wrote related parts of the article. NL annotated and labeled the images used in the experiments and wrote the medical sections of the article. ER consulted significantly for the design of the algorithms. MP consulted for the design of the system. ML was responsible for handling and preprocessing of image data in the virtual microscope environment. HJ was the principal investigator of the FinProg study. JL was responsible for designing the multidisciplinary research setting as a whole. ER and JL contributed also by improving the article. All authors read and approved the final manuscript.
